# Professional development, lifestyle behaviors, and job satisfaction: the mediating role of work-life balance in a multi-sector sample

**DOI:** 10.3389/fpsyg.2026.1923493

**Published:** 2026-10-22

**Authors:** Zhijia Yang

**Affiliations:** School of Marxism, Henan University, Kaifeng, China

**Keywords:** employee training, job satisfaction, lifestyle behaviors, structural equation modeling, work-life balance

## Abstract

**Background:**

Modern organizations face increasing pressure to enhance employee satisfaction and productivity. Although prior research has examined individual antecedents of job satisfaction, less is known about whether professional-development, behavioral, and lifestyle-related resources are associated with job satisfaction through work–life balance.

**Objective:**

This study examines the associations of social media engagement (SME; hereafter used to denote social media engagement), employee training (ET), healthy dietary habits (HDH), online learning participation (OLP), and physical activity (PA) with job satisfaction (JS), with work–life balance (WLB) evaluated as a model-based indirect pathway.

**Methods:**

A quantitative, cross-sectional survey was administered to 437 working professionals across multiple industries. Structural equation modeling (SEM) and bootstrapped indirect-effect analysis were used to assess the measurement model, structural relationships, and indirect associations.

**Results:**

The measurement model demonstrated strong reliability and validity. All five antecedent variables were significantly and positively associated with WLB, with OLP showing the strongest path coefficient (β = 0.379). WLB was also positively associated with JS (β = 0.624, *p* < 0.001). The SRMR value indicated adequate approximate model fit (SRMR = 0.053), whereas the NFI value (0.893) was slightly below the ideal 0.90 threshold and was therefore interpreted cautiously. Bootstrapped indirect-effect analysis supported WLB as a statistically significant indirect pathway linking the antecedent variables with JS.

**Conclusion:**

The findings suggest that professional-development, behavioral, and lifestyle-related resources are associated with job satisfaction through employees' perceived work–life balance. Because the study is cross-sectional, the results should be interpreted as statistical associations rather than evidence of causal effects.

## 1 Introduction

Employee job satisfaction (JS) has become a major concern in the modern competitive workplace, as organizations aim to improve productivity, efficiency, and long-term sustainability (Chen et al., 2022; Udin, 2023). JS reflects employees' overall contentment with their work role, and this has a direct effect on motivation, performance, commitment, and retention (Puiu et al., 2025; Zhang et al., 2024). Employees with higher job satisfaction are more likely to demonstrate stronger performance, more positive workplace relationships, and greater innovative behavior. Due to its great influence on the individual and the organizational performance, JS has been intensively researched in management, psychology, and organizational behavior (Nurhasanah et al., 2023; Yusnita et al., 2022). Nonetheless, JS is a multidimensional construct that depends on several factors, including salary and benefits, working conditions, career growth, job security, leadership, workload, and relationships with co-workers (Ogunola, 2022; Dias et al., 2022).

Work-life balance (WLB) has become increasingly important as employees face growing work demands and pressures associated with technological advancement, digital connectivity, globalization, and changing workplace expectations. A recent review explicitly describes contemporary workplaces as shaped by rapid technological advancement, globalization, and changing societal expectations, making WLB increasingly important for employee wellbeing and organizational outcomes (Bello et al., 2024). Digital technologies can blur work-non-work boundaries, create an “always-on” expectation, increase information overload, and make psychological detachment from work more difficult (Kumar et al., 2024). Leong et al. similarly found that greater digital-technology use was associated with greater work-life conflict (Leong et al., 2025). Sulistiyani et al. (Sulistiyani and Nuphanudin, 2026) identified excessive workload and digital-connectivity demands as important challenges to employees' WLB. Beekman et al. (Beekman et al., 2025) also found that greater telework was associated with increased work pressure and reduced role clarity. WLB can be understood as employees' perceived ability to manage work responsibilities alongside family, health, and social activities (Susanto et al., 2022; Maharani and Tamara, 2024; Çobanoglu et al., 2023). A favorable balance may enhance motivation, psychological health, and JS, whereas poor balance may contribute to stress, burnout, and reduced performance. Thus, WLB is important for understanding employee behavior and overall JS (Lau and Marianti, 2024; Abuhammad et al., 2025; Berger Ploszaj et al., 2025).

Because JS is multidimensional and shaped by interrelated professional, behavioral, and lifestyle factors, advanced multivariate methods are required to examine these relationships simultaneously (Quines and Arendain, 2023; Hemsworth et al., 2026). Structural equation modeling (SEM) is suitable for this purpose because it enables researchers to estimate multiple relationships among latent constructs within a single analytical framework while accounting for measurement error (Alkubati et al., 2025; Bing and Wan, 2025). In the present study, partial least squares structural equation modeling (PLS-SEM) is appropriate because the proposed model includes several antecedent constructs [i.e., social media engagement (SME), employee training (ET), healthy dietary habits (HDH), online learning participation (OLP), and physical activity (PA)] that are examined simultaneously in relation to WLB and JS. PLS-SEM also allows the measurement model and structural model to be assessed together, making it suitable for testing the proposed mediating role of WLB within an integrated framework (Assyahidah et al., 2024; Ciavolino et al., 2024).

ET and OLP were selected because contemporary employees are expected to update their competencies continuously, and training and online learning may strengthen their perceived ability to meet job demands. HDH and PA were included because health-related routines can support employees' physical energy, psychological recovery, and stress regulation, which are relevant to managing work and non-work roles. SME was included because digital-social interaction has become a common feature of professional life: it may support communication, networking, and social support, although it may also intensify boundary pressure when digital engagement becomes excessive. Together, these five antecedents capture distinct but interrelated professional, lifestyle, and behavioral-social conditions through which employees may experience WLB and JS.

To maintain terminological consistency, the term professional-development resources is used in this study as an umbrella term for work-related learning resources that may strengthen employees' competencies and perceived capacity to meet job demands. Within this category, employee training (ET) refers specifically to structured training opportunities provided or supported by the organization, whereas online learning participation (OLP) refers to employees' participation in flexible digital or online learning activities, including self-directed and continuing professional learning. Therefore, ET and OLP are treated as related but distinct professional-development resources. The term lifestyle-related personal resources refers to HDH and PA, because both constructs represent health-related behaviors that may support energy, recovery, and stress regulation. The term behavioral-social resource refers to SME, because social-media engagement may provide communication, professional networking, and social-support functions in digital work life.

This selection is consistent with contemporary organizational behavior frameworks, particularly resource-based perspectives such as the Job Demands-Resources (JD-R) model, which suggest that JS is shaped not only by workplace conditions (Thapa et al., 2025), but also by job-related and personal resources that contribute to employee wellbeing (Swaidan and Jabbour Al Maalouf, 2025; Romero-Carazas et al., 2024). According to JD-R theory, adequate job and personal resources can help employees cope with job demands, support wellbeing, and improve job-related attitudes (Claes et al., 2023; Zhang and Duan, 2023). In this study, ET and OLP are treated as professional-development resources because they may strengthen employees' competencies and perceived ability to meet job demands (Fernández-Salinero et al., 2026). HDH and PA are treated as lifestyle-related personal resources because health-related routines may support energy, recovery, stress regulation, work satisfaction, and WLB (Brailovskaia et al., 2024). SME is treated as a behavioral-social resource because communication with colleagues and professional networks may facilitate social connection and support (Pekkala et al., 2025), which can contribute to employees' WLB (Buonomo et al., 2024). These resources are theoretically connected because each may help employees manage work demands, preserve personal wellbeing, and reduce interference between work and non-work roles. WLB is therefore positioned as the central mechanism through which these resource domains are associated with JS.

Despite this growing body of evidence, prior research has generally treated professional development, lifestyle behaviors, digital-social interaction, WLB, and JS as separate or partially connected research streams. This separation limits the ability to explain whether different resource domains operate through a common work–life balance mechanism. Therefore, the present study examines SME, ET, HDH, OLP, and PA as antecedents of WLB and evaluates whether WLB serves as an indirect pathway linking these antecedents with JS within an integrated SEM framework.

The study's novelty lies in framing WLB as a shared mechanism through which professional-development, lifestyle-related, and behavioral-social resources are associated with JS.

### 1.1 Research objective

This study aims to examine the associations of SME, ET, healthy dietary habits (HDH), online learning participation (OLP), and PA with WLB among working professionals. It further aims to assess the association between WLB and JS, and to test whether WLB serves as an indirect pathway linking these professional-development, behavioral, and lifestyle factors and JS. Finally, the study evaluates these relationships within an integrated SEM framework using a multi-sector sample of working professionals.

### 1.2 Theoretical contributions

This study contributes to organizational psychology by extending the JD-R-informed explanation of JS beyond conventional workplace-centered resources. Specifically, it treats ET and OLP as two distinct professional-development resources, HDH and PA as lifestyle-related personal resources, and SME as a behavioral-social resource shaped by digital workplace interaction. The theoretical contribution is not the mere inclusion of multiple predictors, but the development of a cross-domain resource framework in which WLB functions as the central mechanism connecting these resource domains with JS. This extends JD-R theory in two ways. First, it broadens the resource concept by incorporating resources that extend beyond formal job design, including structured organizational training, flexible online learning, health-related routines, and digital-social interaction. Second, it positions WLB as a boundary-regulation mechanism through which heterogeneous resources may be translated into more favorable job-related attitudes. The use of a multi-sector sample further supports the relevance of this theoretical extension across heterogeneous workplace contexts.

## 2 Literature review

To maintain theoretical coherence, this literature review is organized around two guiding theoretical perspectives: JD-R theory and work–life balance theory. JD-R theory is used to explain how professional-development, lifestyle-related, and behavioral-social resources may help employees manage work demands, while work–life balance theory is used to explain how employees' ability to manage work and non-work roles may be linked to job satisfaction. Therefore, the review is limited to the constructs included in the proposed model: SME, ET, HDH, OLP, PA, WLB, and JS.

### 2.1 JD-R theory, work–life balance theory, and job satisfaction

JD-R theory provides the main theoretical foundation for explaining how resources are associated with employees' wellbeing and job-related attitudes. According to this perspective, job demands consume employees' psychological and physical resources, whereas job and personal resources support coping, motivation, goal attainment, and wellbeing. Claes et al. (Claes et al., 2023) found that job resources were positively associated with employees' subjective wellbeing, whereas job demands were negatively associated with wellbeing; importantly, job satisfaction partially explained these relationships. Zhang and Duan (Zhang and Duan, 2023) similarly demonstrated that job demands negatively predicted postdoctoral job satisfaction, whereas job resources positively predicted satisfaction and reduced burnout risk. These findings support the central JD-R assumption that employees' job-related attitudes cannot be explained only by demands; the resources available to employees must also be considered.

Recent JD-R-informed research also suggests that resources may originate from domains beyond formal job design. Demerouti (Demerouti, 2025) emphasized that JD-R theory remains useful for explaining employee wellbeing and future job design because it allows both job demands and job resources to be examined within a flexible theoretical structure. Yu et al. (2026) further showed that different configurations of job resources can contribute to job satisfaction even when employees experience work–family conflict and social isolation. In the present study, this resource-based logic is extended to three domains that are directly linked to the proposed model: professional-development resources, lifestyle-related personal resources, and behavioral-social resources. ET and OLP are therefore positioned as development resources, HDH and PA as personal health-related resources, and SME as a behavioral-social resource embedded in digital work life.

Work–life balance theory complements JD-R theory by explaining how employees manage the interface between work and non-work roles. WLB refers to employees' perceived ability to manage work responsibilities alongside family, health, and social roles (Susanto et al., 2022; Maharani and Tamara, 2024; Çobanoglu et al., 2023). Prior research has consistently shown that WLB is positively associated with JS. Susanto et al. (2022) demonstrated that WLB was linked to job satisfaction and job performance among SME employees. Çobanoglu et al. (2023) showed that WLB had a mediating role in the relationship between job stress and career satisfaction. Lau and Marianti (Lau and Marianti, 2024) further indicated that work–life balance dimensions were associated with job satisfaction and turnover intention. These findings justify treating WLB as a central construct in explaining employees' job-related attitudes.

The relevance of WLB is particularly important in contemporary work contexts where workload, flexibility, digital connectivity, and boundary management shape employees' daily experiences. Bellini et al. (Mohammed et al., 2022) showed that cognitive demands can increase workload and work–life conflict, while organizational support may not always fully offset these pressures. Andrlić et al. (2024) similarly demonstrated that WLB plays an important role in connecting organizational support with employee work-related outcomes. Jabbar et al. (Raisa et al., 2025) further found that flexible working hours can improve WLB and, through improved balance, contribute to higher JS. Alnagbi et al. (2025) also showed that workload negatively affects WLB, whereas institutional support and digital infrastructure can contribute positively to balance. Together, these studies support the argument that WLB should not be treated only as a separate employee outcome, but also as a mechanism through which resources may be linked to JS. Mohammed et al. (2022) examined indirect mechanisms involving JS and employee behavior in predicting service performance, while Rajendran et al. (2025) showed that employee engagement mediates the relationship between WLB, digital competence, and retention.

Despite this evidence, prior studies have mostly emphasized workplace-centered resources such as workload, flexibility, organizational support, and digital infrastructure. Less attention has been given to how resources from different domains of employees' lives may operate together through WLB. This is important for the present study because SME, ET, HDH, OLP, and PA represent distinct but theoretically connected resources. By organizing these variables through JD-R theory and work–life balance theory, the present model focuses specifically on whether professional-development, lifestyle-related, and behavioral-social resources are associated with JS through WLB.

### 2.2 Employee training as a professional-development resource

In this study, ET is defined as a structured professional-development resource because it refers to organization-provided or organization-supported training that equips employees with knowledge, competencies, and skills needed to meet work requirements. The psychological mechanism linking ET with WLB is primarily competence-based. From a JD-R perspective, training can operate as a job-related resource because it reduces task uncertainty, strengthens role-related confidence, improves perceived capability, and helps employees complete work responsibilities more efficiently (Claes et al., 2023; Zhang and Duan, 2023; Dao et al., 2025; Arthur et al., 2026). When employees feel more competent and better prepared for job demands, they may experience lower work-related strain and less spillover from work into non-work roles. Therefore, ET may contribute to WLB by improving employees' perceived ability to manage job demands within formal organizational expectations. However, because ET is generally initiated, scheduled, or supported by the organization, its WLB benefit depends partly on whether training is integrated into workload planning rather than added as an extra demand.

### 2.3 Online learning participation as a self-directed and flexibility-enhancing resource

OLP is defined as a flexible professional-development resource because it refers to employees' participation in digital, online, or self-directed learning activities that support continuing skill development. Unlike ET, which is typically organization-initiated and embedded in formal training systems, OLP tends to be more self-directed, asynchronous, and adjustable to employees' available time and learning needs. The psychological mechanism linking OLP with WLB is therefore not only competence-based but also autonomy- and control-based. Online learning may strengthen employees' professional capability while also giving them greater discretion over when, where, and how learning occurs (Wu and Wu, 2026; Jayaraman et al., 2023; Zhang et al., 2025). This flexibility may reduce time-boundary pressure, support self-regulation, and help employees align professional development with personal responsibilities. Wu and Wu (2026) showed that continued learning can create additional demands, but adequate time management, institutional support, and coping strategies are critical for maintaining WLB. Therefore, OLP may contribute to WLB when it functions as an autonomy-supportive and flexible learning resource rather than as an additional after-hours obligation. This distinction explains why ET and OLP are treated as related but psychologically distinct professional-development resources in the present model.

### 2.4 Healthy dietary habits as lifestyle-related personal resources

In addition to professional-development resources, employees' lifestyle resources may affect their ability to manage occupational and personal responsibilities. Healthy dietary habits (HDH) contribute to physical functioning and psychological wellbeing and may therefore strengthen employees' capacity to cope with daily demands. Zagzoog et al. (2026) showed that irregular eating patterns, meal skipping, and low fruit and vegetable consumption are associated with higher stress, whereas healthier dietary behaviors are associated with more favorable psychological conditions. Although direct research linking HDH specifically to WLB remains comparatively limited, these findings suggest that healthy eating can operate as a personal health resource that helps employees maintain the physical and psychological capacity required to manage work and non-work roles.

### 2.5 Physical activity as a recovery and wellbeing resource

A stronger body of evidence supports the role of PA as a personal resource. Regular PA can contribute to psychological recovery, physical health, stress regulation, and positive work-related functioning. Brailovskaia et al. (2024) demonstrated experimentally that increasing PA, particularly when combined with reduced non-work smartphone use, improved employees' work satisfaction, motivation, WLB, positive mental health, and perceived control. Dere et al. (2026) similarly recognizes PA as an important behavioral pathway connecting supportive work environments with employee attitudes and outcomes. These findings provide support for treating PA as a lifestyle-related personal resource capable of contributing positively to WLB. In addition to professional-development and lifestyle-related resources, the model also includes SME as a behavioral-social resource because digital communication can shape employees' access to social support and boundary management.

### 2.6 Social media engagement as a boundary- and recovery-relevant behavioral-social resource

In this study, SME refers to social media engagement as a behavioral-social construct, rather than to small and medium-sized enterprises. SME represents a distinct type of resource because its consequences for WLB depend on how digital communication is used and how employees manage boundaries between work and non-work roles. From a boundary-theory perspective, social media can weaken work–non-work boundaries when it creates constant availability expectations, after-hours interruptions, or competing role demands (Ashforth et al., 2000; Clark, 2000). However, when social media is used for professional networking, coworker communication, information exchange, and social support, it may help employees manage role transitions more smoothly and maintain greater perceived control over work-related demands. From a recovery-theory perspective, digital engagement may undermine recovery when it prevents psychological detachment, but it may also support recovery indirectly when it provides emotional support, reduces uncertainty, and helps employees resolve work-related concerns more efficiently (Sonnentag and Fritz, 2007). Therefore, the positive hypothesis in this study is not based on the assumption that all forms or intensities of social media use are beneficial. Rather, it reflects the resource-oriented function of SME as operationalized in this study, particularly its role in professional connection and perceived support. This dual role also suggests that future research should examine non-linear effects and boundary-related moderators, such as after-hours availability expectations, digital overload, segmentation preference, and perceived boundary control.

### 2.7 Research gap

Although the reviewed literature supports the relevance of professional-development, lifestyle-related, and behavioral-social resources, existing evidence remains fragmented in three main ways. First, many studies on WLB and JS have been conducted in specific occupational groups or restricted organizational contexts. Jayaraman et al. (2023) examined construction workers, Santillan et al. (2023) focused on employees in a technology-company hybrid work context, and Bashir et al. (2025) examined academic employees. Similarly, Alnagbi et al. (2025) focused on female faculty members, Wu and Wu (2026) examined university teachers pursuing further education, Zhang et al. (2025) examined online continuing education among midwives, and Sokolović et al. (2023) focused on media organizations. These studies provide useful evidence, but their occupational or sectoral concentration limits understanding of whether the same resource–WLB–JS logic applies across a more heterogeneous multi-sector sample.

Second, prior studies have usually examined a limited set of antecedents rather than integrating professional-development, lifestyle-related, and behavioral-social resources within one theoretical framework. Bellini et al. (2025) emphasized cognitive demands, workload, flexible work, and social support; Andrlić et al. (2024) focused on organizational support and WLB; Jabbar et al. (Raisa et al., 2025) examined flexible working hours and WLB; and Alnagbi et al. (2025) examined workload, institutional support, and digital infrastructure. These studies clarify the importance of workplace-centered resources, but they provide less explanation of how resources originating from different domains of employees' lives may jointly contribute to WLB and JS.

Third, the antecedents included in the present model have largely developed as separate research streams. Dao et al. (2025) and Sokolović et al. (2023) examined employee training and professional development, whereas Wu and Wu (2026), Xiang and Yu (2025), and Zhang et al. (2025) focused on online learning and continuing professional education. In contrast, Zagzoog et al. (2026), Sonnentag et al. (2023), and Jackson et al. (2025) examined healthy eating and health-related behaviors, while Brailovskaia et al. (2024) and Dere et al. (2026) emphasized physical activity and employee wellbeing. Research on SME has also developed separately, with Pekkala et al. (2025) and Fusi and Zhang (2020) focusing on social-media communication, coworker community, and professional interaction. This separation creates more than an empirical gap; it creates a theoretical gap in explaining how heterogeneous resources may be connected to job-related attitudes through a common WLB mechanism.

From a JD-R perspective, this gap is important because employee resources are often treated as workplace-centered resources, while fewer studies examine how professional-development resources, lifestyle-related personal resources, and behavioral-social resources may jointly contribute to employees' WLB and JS. Addressing this gap can extend organizational psychology by clarifying whether WLB functions as a boundary-regulation and resource-conversion mechanism through which heterogeneous resources are translated into job-related satisfaction. Therefore, the present study examines SME, ET, HDH, OLP, and PA as antecedents of WLB and evaluates whether WLB serves as a shared indirect pathway linking these antecedents with JS.

### 2.8 Novelty of the study

The novelty of this study does not rest only on combining several antecedent variables, but on extending the JD-R-informed explanation of JS toward a cross-domain resource-mechanism framework. This novelty is justified by prior evidence showing that WLB–JS studies have often been conducted in specific occupational contexts (Alnagbi et al., 2025; Jayaraman et al., 2023; Santillan et al., 2023; Bashir et al., 2025), while research on training, online learning, health behaviors, physical activity, and SME has generally developed in separate streams (Brailovskaia et al., 2024; Pekkala et al., 2025; Dao et al., 2025; Wu and Wu, 2026). Traditional applications of JD-R theory have primarily emphasized job demands and job resources located within the workplace. In contrast, the present study conceptualizes employee resources as extending across three interconnected domains: formal professional-development resources, lifestyle-related personal resources, and behavioral-social resources embedded in digital work life. The theoretical novelty therefore lies in positioning WLB as a boundary-regulation and resource-conversion mechanism through which these heterogeneous resources are associated with JS. This framing advances prior WLB–JS research by explaining not only whether WLB is associated with JS, but how different resource domains may converge through WLB as a shared indirect pathway. Accordingly, the study contributes to JD-R theory by broadening the meaning of employee resources beyond conventional workplace supports and by clarifying WLB as the mechanism through which professional-development, lifestyle-related, and digital-social resources are linked to job-related attitudes.

### 2.9 Hypotheses development and conceptual model

Building on JD-R theory and work–life balance theory, this study conceptualizes ET and OLP as two related but distinct professional-development resources: ET refers to structured organizational training, whereas OLP refers to flexible digital or online learning (Fernández-Salinero et al., 2026; Dao et al., 2025; Arthur et al., 2026; Wu and Wu, 2026), HDH and PA are conceptualized as lifestyle-related personal resources because they represent health-related behaviors that may support energy, recovery, and stress regulation (Brailovskaia et al., 2024; Zagzoog et al., 2026; Dere et al., 2026; Sonnentag et al., 2023; Jackson et al., 2025). SME is conceptualized as a behavioral-social resource because digital engagement may provide social support, professional networking, and communication benefits, while excessive connectivity may also create boundary pressure (Kumar et al., 2024; Leong et al., 2025; Pekkala et al., 2025; Fusi and Zhang, 2020). The positive theoretical rationale for H1 is grounded in boundary theory (Ashforth et al., 2000; Clark, 2000), recovery theory (Sonnentag and Fritz, 2007), and workplace social-support literature. From a boundary perspective, social media may support WLB when it helps employees coordinate work demands, maintain professional networks, and access support without substantially increasing role intrusion. From a recovery perspective, supportive digital interaction may reduce uncertainty and provide emotional or informational resources that help employees manage work-related concerns more efficiently. Social-media communication can also help employees maintain coworker community, information exchange, and interpersonal support (Pekkala et al., 2025; Fusi and Zhang, 2020), while perceived support from colleagues and leaders has been linked to better WLB (Buonomo et al., 2024). Therefore, H1 tests the resource-oriented association between SME and WLB as specified in the present model, while recognizing that excessive or intrusive digital connectivity may intensify work–non-work boundary conflict (Kumar et al., 2024; Leong et al., 2025). ET is expected to support WLB mainly through a structured competence-building mechanism: organization-supported training may reduce task uncertainty, strengthen job-related confidence, and improve employees' perceived capacity to meet formal work demands (Fernández-Salinero et al., 2026; Dao et al., 2025; Arthur et al., 2026). In contrast, OLP is expected to support WLB through both competence-building and autonomy-control mechanisms. Because online learning is often self-directed, asynchronous, and accessible across time and location, it may allow employees to develop new skills while maintaining greater control over the timing and intensity of learning activities (Wu and Wu, 2026; Zhang et al., 2025; Xiang and Yu, 2025). Thus, ET and OLP are treated as related professional-development resources, but their psychological pathways differ: ET primarily reflects organizational support for capability development, whereas OLP additionally reflects flexibility, perceived autonomy, and self-regulated learning.

HDH may support WLB by strengthening employees' physical energy and stress-coping capacity (Zagzoog et al., 2026; Sonnentag et al., 2023; Jackson et al., 2025), while PA may improve WLB through recovery, stress regulation, psychological wellbeing, and work satisfaction (Brailovskaia et al., 2024; Dere et al., 2026). Finally, WLB is specified as a model-based indirect pathway because prior studies show that WLB links workplace resources, employee wellbeing, and JS (Susanto et al., 2022; Çobanoglu et al., 2023; Raisa et al., 2025). Based on this theoretical foundation, the following hypotheses are proposed:

**H1**. SME is positively associated with employees' WLB.
**H2**. ET has a significant positive association with employees' WLB.
**H3**. HDH have a significant positive association with employees' WLB.
**H4**. OLP has a significant positive association with employees' WLB.
**H5**. PA has a significant positive association with employees' WLB.
**H6**. WLB serves as a significant model-based indirect pathway linking the antecedent variables with employees' JS.

**a**. WLB serves as a significant indirect pathway linking SME with JS.
**b**. WLB serves as a significant indirect pathway linking ET with JS.
**c**. WLB serves as a significant indirect pathway linking HDH with JS.
**d**. WLB serves as a significant indirect pathway linking OLP with JS.
**e**. WLB serves as a significant indirect pathway linking PA with JS.

Figure 1 presents the conceptual model. The model groups the antecedents into three resource domains: behavioral-social resource (SME), professional-development resources (ET and OLP), and lifestyle-related personal resources (HDH and PA). These antecedents are specified as associated with WLB, which is then specified as an indirect pathway linking the antecedents with JS. Consistent with the cross-sectional design, the proposed paths represent theoretically specified associations rather than causal effects (Claes et al., 2023; Zhang and Duan, 2023; Dao et al., 2025).

**Figure 1:**
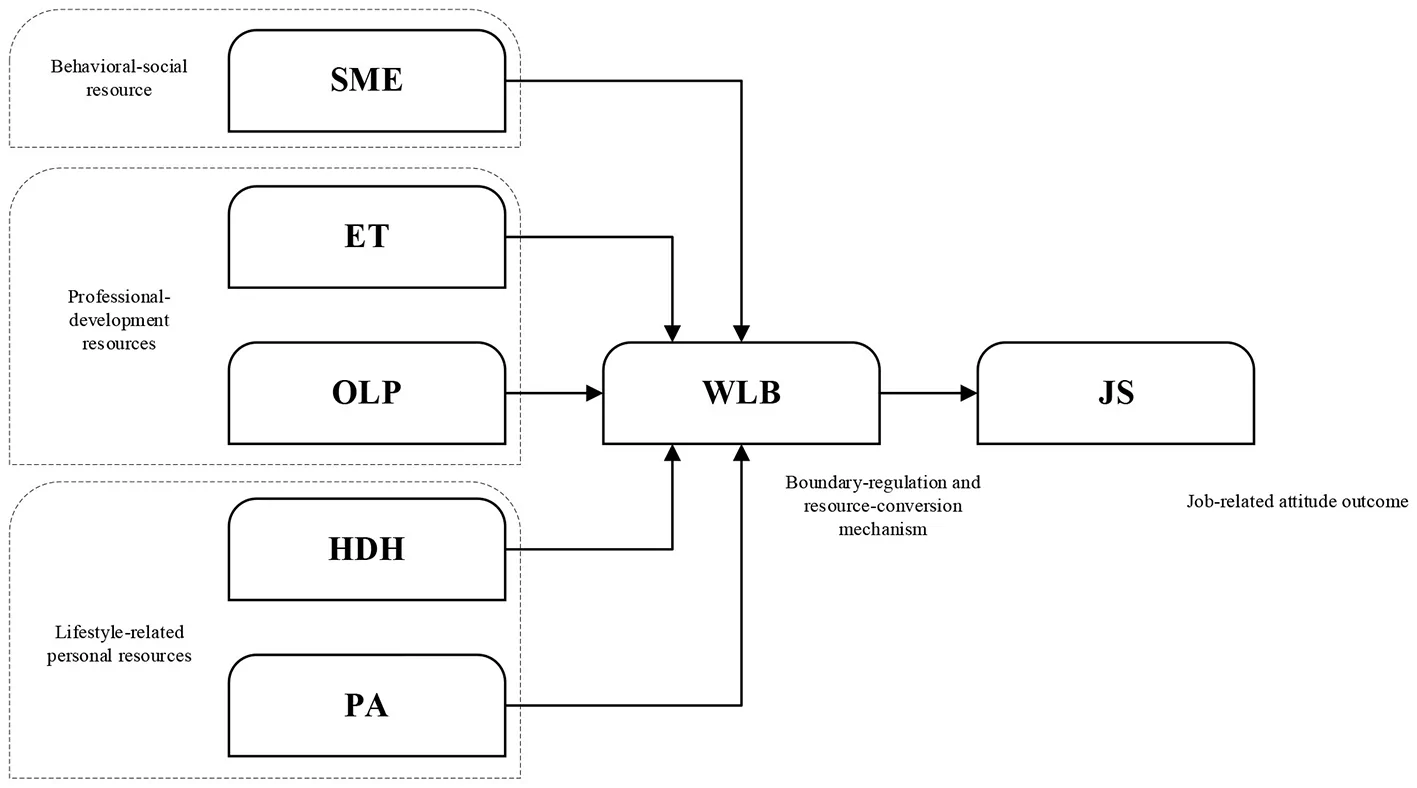
Conceptual model linking professional-development, lifestyle-related, and behavioral-social resources with JS through WLB.

## 3 Methodology

The study used a quantitative cross-sectional design based on survey data collected from 437 working professionals across different industries. The variables measured through structured questionnaire items were JS, WLB, SME, ET, HDH, OLP, and PA. Because the data were collected at a single point in time, the findings are interpreted as statistical associations rather than causal effects. The proposed conceptual model was evaluated using PLS-SEM, including assessment of the measurement model, structural paths, and bootstrapped indirect effects. SPSS was used only for data screening, coding verification, missing-data inspection, and descriptive statistics. The SEM phase was conducted using PLS-SEM procedures to assess the measurement model, structural model, and bootstrapped indirect effects. Therefore, final hypothesis decisions were based on the PLS-SEM structural model and bootstrapped indirect-effect results, not on separate SPSS-based correlation, regression, *t*-test, or ANOVA analyses.

### 3.1 Data collection

This study used a structured questionnaire survey to collect data from working professionals across multiple employment sectors, including information technology, education, healthcare, finance, manufacturing, and service industries. The questionnaire was distributed online through professional networks, email invitations, and organizational contacts to ensure diverse participation. A non-probability purposive-convenience sampling strategy was used because the target population consisted of working professionals across multiple sectors and no complete sampling frame was available for probability sampling. Participants were recruited based on eligibility criteria, including full-time employment status and at least 1 year of work experience. This approach enabled access to respondents from diverse occupational sectors, but it may also introduce selection bias because participants were not randomly selected. In addition, online distribution may create coverage bias and self-selection bias, as individuals with stronger interest in workplace wellbeing, digital learning, professional development, or lifestyle behaviors may have been more likely to participate. Therefore, the sample provides useful multi-sector variation but should not be interpreted as statistically representative of all working professionals.

A total of 520 questionnaires were distributed, and 462 responses were received, yielding a response rate of 88.8%. After removing incomplete and inconsistent entries, 437 valid responses were retained for analysis, corresponding to an effective valid response rate of 84.0%. Only full-time employees with at least 1 year of work experience were included, while students, part-time workers, and incomplete responses were excluded. Participation was voluntary, and respondents were informed about the academic purpose of the study. The final sample provided a sufficiently large and diverse dataset suitable for structural equation modeling analysis. Data cleaning was conducted to eliminate unresponsive data, missing data and duplications. Incomplete questionnaires were excluded from the final dataset. The data after cleaning were in the form of 437 valid responses that were subjected to statistical analysis.

### 3.2 Demographic characteristics

Table 1 displays the respondents' demographic characteristics. Participants were chosen from a variety of industries, representing a range of age groups, genders, and work experience levels. The diversity of the sample supports the suitability of the data for examining JS and WLB among working professionals.

**Table 1:** The demographic profile of the participants (*N* = 437).

Variable	Category	Number of participants	Percentage (%)
Gender	Female	210	48%
	Male	227	52%
Age	21–30	201	46%
	31–40	149	34%
	41+	87	20%
Work Experience	1–5 years	184	42%
	6–10 years	136	31%
	10+ years	117	27%
Sector	IT	102	23.3%
	Education	88	20.1%
	Healthcare	74	16.9%
	Finance	65	14.8%
	Manufacturing	54	12.4%
	Service	54	12.4%

### 3.3 Questionnaire

The questionnaire was constructed by adapting measurement items from prior studies on organizational behavior, employee wellbeing, professional development, lifestyle behavior, work–life balance, and job satisfaction. The instrument measured seven latent constructs: SME, ET, HDH, OLP, PA, WLB, and JS. All items were measured using a five-point Likert scale ranging from 1 = strongly disagree to 5 = strongly agree. The items were phrased as statements to ensure consistency with Likert-scale measurement and were refined through literature review, expert assessment, and pre-testing before the final survey was distributed.

The questionnaire-development process involved three steps. First, relevant constructs and candidate indicators were identified from prior empirical and theoretical studies. Second, the wording of the items was adapted to fit the context of working professionals across multiple sectors while preserving the conceptual meaning of the original constructs. Third, the questionnaire was reviewed for clarity, relevance, and construct alignment before data collection. Content validity was addressed by ensuring that each item corresponded directly to the conceptual definition of its intended construct. Construct validity was subsequently assessed through the PLS-SEM measurement model using indicator loadings, average variance extracted (AVE), composite reliability (CR), Cronbach's alpha, variance inflation factor (VIF), and discriminant validity tests. The questionnaire is shown in Table 2.

**Table 2:** The questionnaire statements list.

Construct	Indicator	Statement
ET	ET1	My organization provides training programs regularly.
	ET2	Training programs in my organization help me improve my job performance.
	ET3	Training programs enhance my professional skills and career development.
SME	SME1	I use social media regularly during a typical day.
	SME2	Social media use affects my daily routine.
	SME3	Social media helps me stay connected with professional networks.
HDH	HDH1	My food habits help me stay energetic at work.
	HDH2	I avoid unhealthy food during working hours.
	HDH3	I maintain healthy eating habits during workdays.
OLP	OLP1	I am interested in continuous online learning.
	OLP2	I spend time learning new skills through online platforms.
	OLP3	Online learning contributes to my professional growth.
PA	PA1	Physical activity helps me reduce stress.
	PA2	I feel physically active during most days of the week.
	PA3	I regularly engage in exercise or fitness activities.
WLB	WLB1	I can manage my work responsibilities well.
	WLB2	I can manage my personal and professional responsibilities without excessive stress.
	WLB3	I am satisfied with the balance between my work and personal life.
JS	JS1	I am satisfied with my current job.
	JS2	I feel valued in my current workplace.
	JS3	I would recommend my organization as a good place to work.

Each latent construct was measured using three indicators adapted from prior organizational behavior and employee wellbeing studies, which is illustrated in Table 3. The use of three indicators per construct is consistent with SEM recommendations for achieving adequate construct reliability and validity.

**Table 3:** Measurement basis and adaptation notes for the questionnaire constructs.

Construct	Measurement source	Adaptation
SME	Workplace social-media communication and professional-networking literature (Pekkala et al., 2025; Fusi and Zhang, 2020)	Adapted to assess frequency/routine of use and professional/social connection
ET	Employee training and professional-development measures (Sokolović et al., 2023)	Adapted to assess training availability, usefulness, skill development, and career development
HDH	Work–healthy-eating and health-behavior measures (Sonnentag et al., 2023; Jackson et al., 2025)	Adapted to assess healthy eating during workdays, healthy food choices, and avoidance of unhealthy eating
OLP	Online learning/continuing professional-development measures (Zhang et al., 2025; Xiang and Yu, 2025)	Adapted to assess interest, participation, and perceived professional development
PA	Physical-activity and work–exercise measurement literature (Sonnentag et al., 2023)	Adapted to assess regularity and level of PA and exercise
WLB	Established WLB measurement literature (Zhang et al., 2024; Nitzsche et al., 2014)	Adapted to assess management and compatibility of work and personal roles and perceived balance
JS	Validated overall job-satisfaction measurement literature (Zhang et al., 2024; Limniou et al., 2025)	Adapted to assess employees' overall satisfaction and positive evaluation of their job

### 3.4 Statistical analysis

The analytical workflow was conducted in two sequential stages. In the first stage, SPSS was used for preliminary data handling, including coding verification, missing-data inspection, descriptive statistics, and basic data screening. These analyses were used only to prepare and inspect the dataset and were not used as the basis for hypothesis testing. In the second stage, the proposed model was evaluated using PLS-SEM. The PLS-SEM analysis included assessment of the reflective measurement model using indicator loadings, internal consistency reliability, composite reliability, average variance extracted (AVE), and discriminant validity using the HTMT criterion. The structural model was then evaluated using path coefficients, *t*-values, *p*-values, bootstrapping, *R*^2^ and adjusted *R*^2^ values for endogenous constructs, *f*^2^ effect sizes for direct structural paths, and *Q*^2^ values obtained through blindfolding to assess predictive relevance. Final decisions regarding H1–H6 were based exclusively on the PLS-SEM structural model and bootstrapped indirect-effect results rather than on separate Pearson correlation, regression, *t*-test, or ANOVA analyses (Nitzsche et al., 2014). Indirect effects were assessed using bootstrapped mediation analysis. For each antecedent variable, WLB was specified as the model-based indirect pathway linking the antecedent with JS. Bootstrapped confidence intervals were used to determine whether the indirect effects were statistically significant. This approach is appropriate because PLS-SEM enables the measurement model, structural paths, explanatory power, effect sizes, predictive relevance, and indirect pathways to be evaluated within a single model-based framework (Hair et al., 2021).

### 3.5 Common method bias assessment

Because the study used a single-source, self-reported online questionnaire, common method bias (CMB) was considered a potential methodological concern. Procedural remedies were applied during questionnaire administration by ensuring anonymity, informing respondents that there were no right or wrong answers, using clear and neutral item wording, and separating the measurement of different constructs within the questionnaire. In addition, a statistical diagnostic was conducted using full collinearity VIF assessment, which has been recommended for detecting potential CMB in PLS-SEM models (Kock, 2015). The full collinearity VIF values ranged from 2.841 to 4.794, indicating that severe collinearity was not present under the commonly used VIF < 5 criterion. However, because several values exceeded the more conservative 3.3 threshold, especially OLP (VIF = 4.794) and ET (VIF = 4.733), the possibility of common method influence or partial measurement overlap among professional-development constructs cannot be completely ruled out. Therefore, the findings are interpreted cautiously and CMB is acknowledged as a limitation of the cross-sectional self-report design. The VIF assessment for CMB is illustrated in Table 4.

**Table 4:** Full collinearity VIF assessment for common method bias.

Construct	Full collinearity VIF	Interpretation
SME	3.504	Below 5; above conservative 3.3
ET	4.733	Below 5; above conservative 3.3
HDH	2.841	Below 5
OLP	4.794	Below 5; above conservative 3.3
PA	3.235	Below 5
WLB	3.326	Approximately at conservative threshold
JS	3.121	Below 5

VIF values below 5 indicate no severe full collinearity, while values above the more conservative 3.3 criterion suggest that common method influence should be interpreted cautiously. The relatively high values for OLP and ET may reflect their conceptual proximity as professional-development resources, but they may also indicate some degree of measurement redundancy or method-related inflation resulting from the single-wave self-report design. Because the measurement model showed high internal consistency values, the CMB assessment was considered particularly important to ensure that the observed relationships were not primarily attributable to method-related inflation (Kock, 2015).

## 4 Result

The following section reports the results of the PLS-SEM analysis used to evaluate the proposed conceptual model. The analysis includes measurement-model assessment, discriminant validity, common method bias assessment, structural-model hypothesis testing, explanatory power using *R*^2^ and adjusted *R*^2^, path-specific effect sizes using *f*^2^, predictive relevance using blindfolding-based *Q*^2^, bootstrapped indirect-effect analysis, and model-fit evaluation. SPSS-based procedures were limited to preliminary data screening and descriptive inspection; therefore, hypothesis decisions were based on the PLS-SEM structural model and bootstrapped indirect effects.

### 4.1 Measurement model evaluation

The measurement model was evaluated for reliability and validity, as reported in Table 5 and Figure 2. The VIF values ranged from 2.311 to 4.625 and the outer loadings from 0.890 to 0.950, suggesting strong reliability without multicollinearity. Strong convergent validity and internal consistency were verified by the AVE values (0.807–0.890), composite reliability (CR) (0.926–0.961), and Cronbach's alpha (α) (0.881–0.939). The measurement model thus satisfied every prerequisite for additional structural analysis.

**Table 5:** Measurement model assessment results.

Construct	Item code	Outer Loadings	VIF	AVE	CR		C. Alpha
					CR (rho_a)	CR (rho_C)	
sET	ET1	0.938	3.822	0.883	0.934	0.958	0.934
	ET2	0.942	4.183				
	ET3	0.938	3.794				
SME	SME1	0.949	4.106	0.890	0.949	0.961	0.939
	SME2	0.942	4.260				
	SME3	0.939	4.202				
HDH	HDH1	0.935	3.452	0.862	0.927	0.949	0.920
	HDH2	0.915	3.173				
	HDH3	0.934	3.473				
OLP	OLP1	0.934	3.810	0.884	0.937	0.958	0.934
	OLP2	0.940	3.913				
	OLP3	0.946	4.155				
PA	PA1	0.934	3.693	0.889	0.938	0.960	0.937
	PA2	0.943	4.280				
	PA3	0.950	4.625				
WLB	WLB1	0.891	2.399	0.812	0.886	0.928	0.884
	WLB2	0.899	2.478				
	WLB3	0.912	2.656				
JS	JS1	0.890	2.311	0.807	0.881	0.926	0.881
	JS2	0.894	2.422				
	JS3	0.911	2.680				

**Figure 2:**
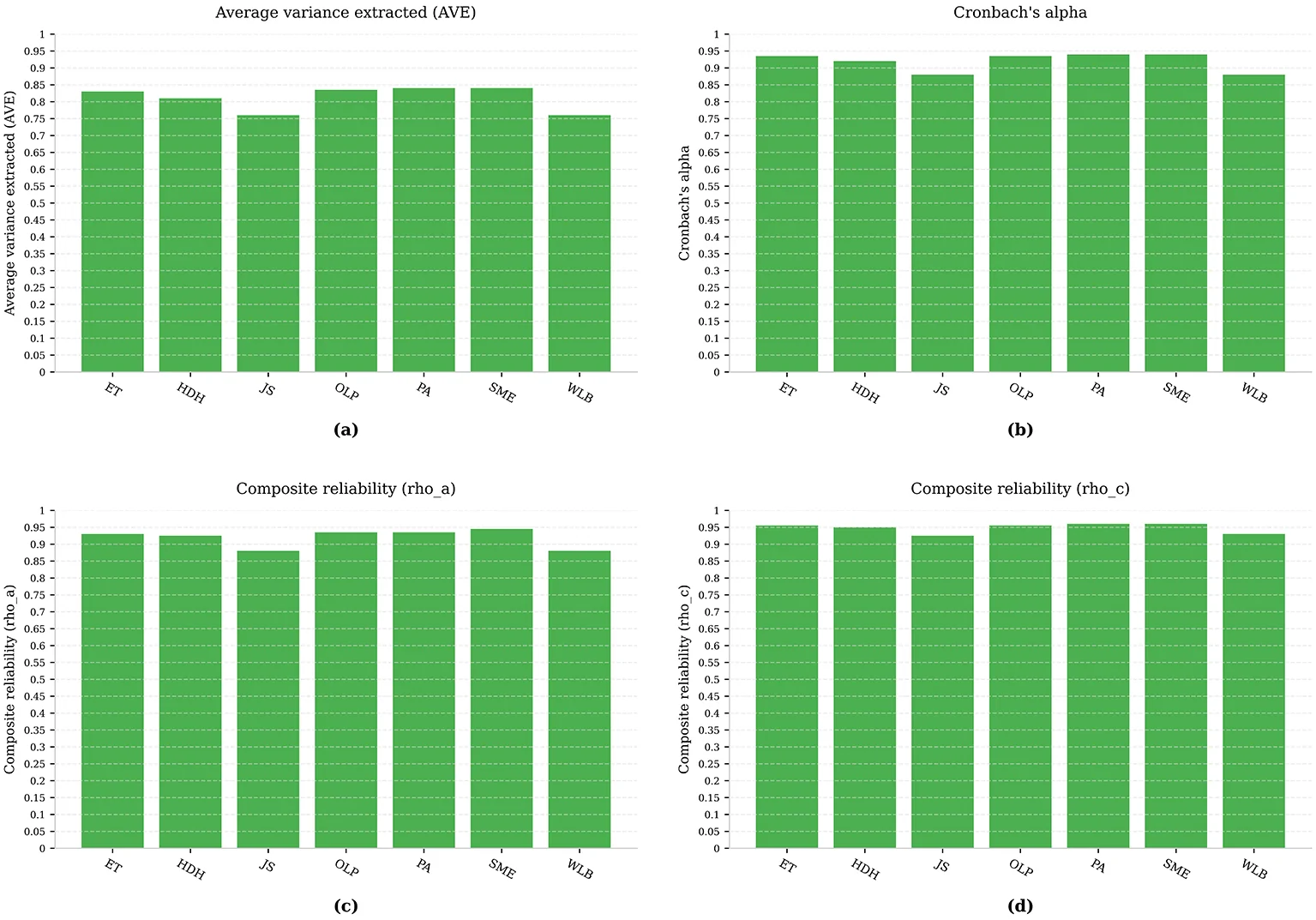
Assessment of measurement model reliability and convergent validity: **(a)** average variance extracted (AVE), **(b)** Cronbach's alpha, **(c)** composite reliability (rho_a), and **(d)** composite reliability (rho_c)

These results provide empirical evidence of construct reliability and convergent validity, as all item loadings exceeded the recommended 0.70 threshold, AVE values exceeded 0.50, and CR and Cronbach's alpha values were above the commonly accepted 0.70 criterion. Therefore, the retained measurement items demonstrated adequate internal consistency and construct validity for subsequent structural-model testing.

Although the reliability values were high, the item loadings, AVE, CR, and Cronbach's alpha values were interpreted together with discriminant-validity evidence, cross-loadings, and common method bias diagnostics to ensure that the results did not merely reflect item redundancy or method-related inflation (Hair et al., 2021; Kock, 2015).

### 4.2 Discriminant validity

Discriminant validity was assessed using three complementary procedures: the Fornell–Larcker criterion, cross-loading inspection, and the Heterotrait–Monotrait ratio (HTMT; Hair et al., 2021; Fornell and Larcker, 1981; Henseler et al., 2015). The Fornell–Larcker criterion was evaluated by comparing the square root of each construct's AVE with its correlations with other constructs. Cross-loadings were inspected to confirm that each indicator loaded more strongly on its intended construct than on the remaining constructs. These additional checks were used to complement the HTMT assessment and provide stronger evidence that the constructs were empirically distinct.

The HTMT criterion was used to assess whether the constructs were empirically distinct within the proposed model. All HTMT values were below the recommended threshold, supporting discriminant validity. This demonstrates that the measurement model is appropriate, and to test the hypotheses, the structural model was assessed further (Table 6 and Figure 3).

**Table 6:** Discriminant validity results using HTMT ratio.

Construct	ET	HDH	JS	OLP	PA	SME	WLB
ET	—						
HDH	0.04	—					
JS	0.427	0.276	—				
OLP	0.037	0.033	0.399	—			
PA	0.039	0.042	0.244	0.015	—		
SME	0.058	0.059	0.192	0.021	0.032	—	
WLB	0.31	0.309	0.706	0.402	0.355	0.377	—

**Figure 3:**
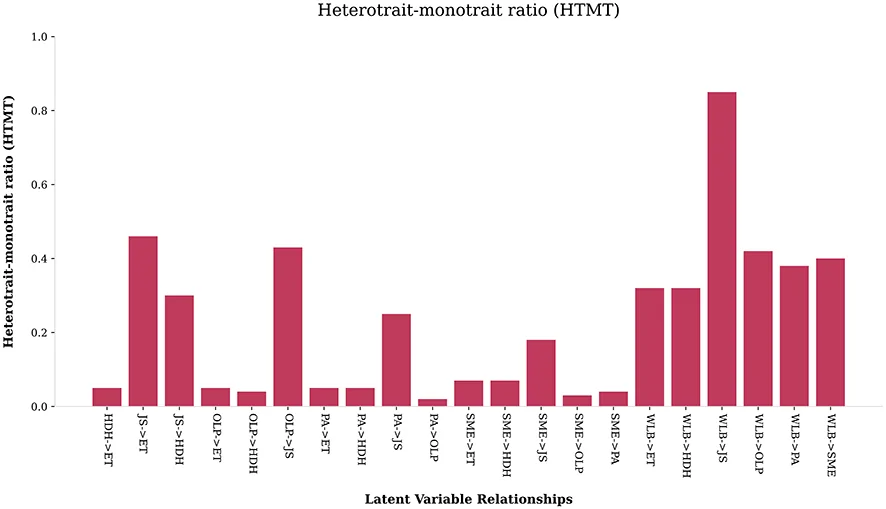
Graphical representation of HTMT values for latent variables.

All HTMT values were below the conservative 0.85 threshold, with the highest value observed for WLB and JS (0.706). These results indicate that the constructs were empirically distinguishable. Together with the Fornell–Larcker and cross-loading checks, the HTMT results support the discriminant validity of the measurement model.

### 4.3 Structural model and hypothesis testing

The structural model was evaluated using PLS-SEM bootstrapping and structural-model quality criteria. Path coefficients, *t*-values, *p*-values, derived standard errors, and approximate confidence intervals were used to assess the statistical significance of the proposed paths. In addition, *R*^2^ and adjusted *R*^2^ were used to assess the explanatory power of the model for the endogenous constructs, *f*^2^ values were used to evaluate the effect size of each direct structural path, and *Q*^2^ values obtained through blindfolding were used to assess predictive relevance. As shown in Table 7, SME, ET, HDH, OLP, and PA were positively associated with WLB, and WLB was positively associated with JS. Therefore, hypothesis decisions were based on the complete PLS-SEM structural model rather than separate correlation, regression, *t*-test, or ANOVA analyses.

**Table 7:** Structural model assessment using PLS-SEM bootstrapping.

Hypothesis	Path	β	t-value	Derived SE	p-value	Approx. 95% CI LL	Approx. 95% CI UL	*f* ^2^	Effect size	Decision
H1	SME → WLB	0.332	10.204	0.033	< 0.001	0.268	0.396	0.27	Medium	Supported
H2	ET → WLB	0.312	9.907	0.031	< 0.001	0.25	0.374	0.238	Medium	Supported
H3	HDH → WLB	0.272	7.969	0.034	< 0.001	0.205	0.339	0.181	Medium	Supported
H4	OLP → WLB	0.379	11.494	0.033	< 0.001	0.314	0.444	0.351	Large	Supported
H5	PA → WLB	0.294	9.711	0.03	< 0.001	0.235	0.353	0.211	Medium	Supported
H6	WLB → JS	0.624	22.902	0.027	< 0.001	0.571	0.677	0.637	Large	Supported

β denotes the standardized path coefficient, and SE denotes the standard error derived from β*/t*. The reported confidence intervals are approximate normal-theory intervals calculated from the available path coefficients and derived standard errors. The *f*^2^ values indicate the path-specific contribution of each predictor to the explained variance of the corresponding endogenous construct. The results show statistically significant structural paths in the proposed model, while the *f*^2^ values provide additional information on the relative practical contribution of each antecedent. Figure 4 illustrates the SEM model diagram.

**Figure 4:**
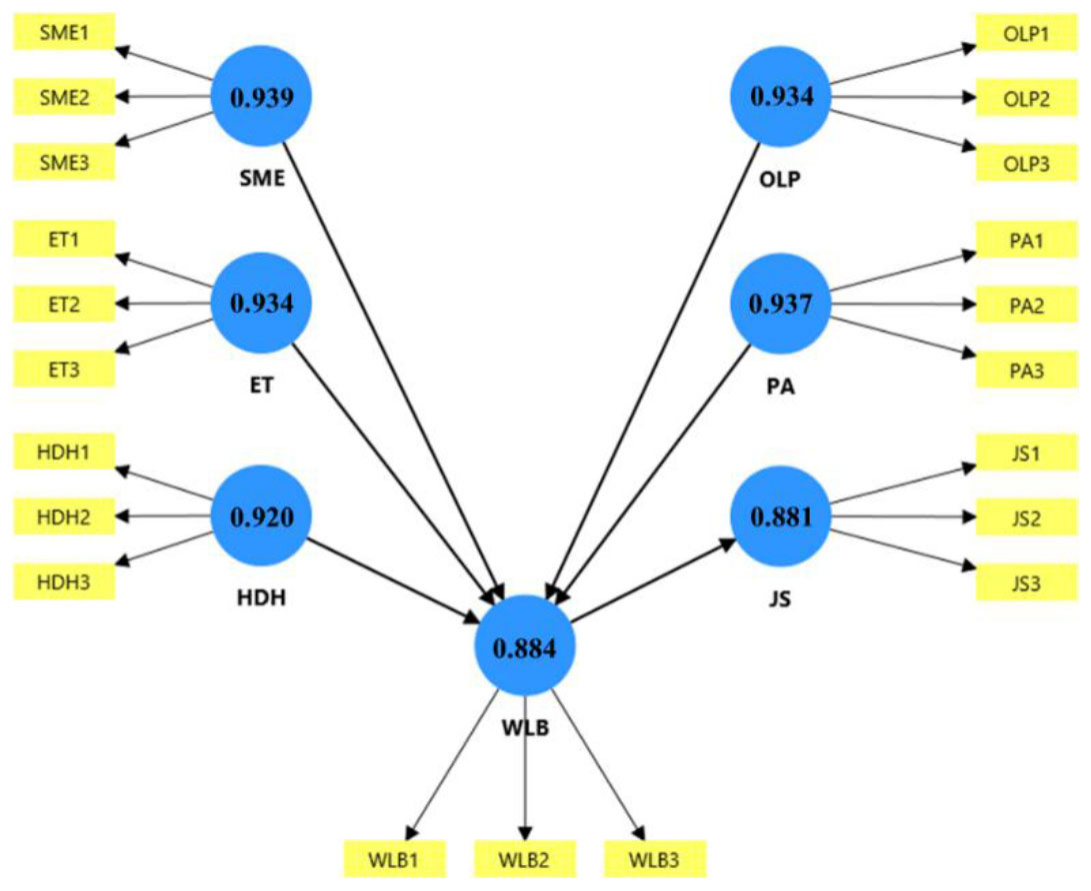
SEM model with WLB as mediator.

Table 8 reports the explanatory power and predictive relevance of the endogenous constructs. The *R*^2^ values indicate the proportion of variance explained in WLB and JS by the proposed structural model, while the adjusted *R*^2^ values account for model complexity. The blindfolding-based *Q*^2^ values were used to evaluate predictive relevance. Positive *Q*^2^ values indicate that the model has predictive relevance for the corresponding endogenous construct. Together, these indices complement the path-significance results by showing the model's explanatory and predictive contribution.

**Table 8:** Explanatory power and predictive relevance of endogenous constructs.

Endogenous construct	*R* ^2^	Adjusted *R*^2^	*Q* ^2^	Interpretation
WLB	0.591	0.588	0.374	The five antecedents explain about 59.1% of the variance in WLB. This is a moderate-to-substantial/large level of explanatory power. Q^2^ > 0 and Q^2^ > 0.35 indicates strong/large predictive relevance.
JS	0.389	0.388	0.257	WLB explains about 38.9% of the variance in JS. This is a moderate level of explanatory power. Q^2^ > 0 and Q^2^ > .15 indicates medium predictive relevance.

### 4.4 Mediation analysis

Bootstrapped mediation analysis was conducted to examine whether WLB served as an indirect pathway linking SME, ET, HDH, OLP, and PA with JS. As shown in Table 9, all five indirect effects were statistically significant because the 95% bootstrapped confidence intervals did not include zero. Specifically, SME, ET, HDH, OLP, and PA were each indirectly associated with JS through WLB. These findings support the mediating role of WLB in the proposed model. However, because the study used a cross-sectional design, the mediation results should be interpreted as model-based indirect associations rather than evidence of causal mediation.

**Table 9:** Bootstrapped indirect effects through work-life balance.

Indirect path	Indirect effect	Boot SE	z/t value	p-value	95% CI LL	95% CI UL	Decision
SME → WLB → JS	0.347	0.035	9.911	< 0.001	0.279	0.417	Significant
ET → WLB → JS	0.266	0.03	8.745	< 0.001	0.207	0.327	Significant
HDH → WLB → JS	0.257	0.029	8.921	< 0.001	0.201	0.313	Significant
OLP → WLB → JS	0.259	0.032	8.201	< 0.001	0.199	0.322	Significant
PA → WLB → JS	0.304	0.032	9.51	< 0.001	0.243	0.367	Significant

The indirect-effect results support WLB as a statistically significant model-based indirect pathway linking SME, ET, HDH, OLP, and PA with JS. However, because the available structural model did not simultaneously estimate direct paths from SME, ET, HDH, OLP, and PA to JS, the findings cannot distinguish full mediation from partial mediation. Accordingly, the results are interpreted as supporting an indirect-only theoretical specification rather than as empirical proof of full mediation. Because the study used a cross-sectional design, the mediation findings should be understood as model-based indirect associations rather than evidence of causal transmission.

### 4.5 Model fit analysis

Table 10 presents the SEM model fit indices. The estimated SRMR value was 0.053, which is below the commonly used 0.08 threshold and indicates adequate approximate model fit. The NFI value for the estimated model was 0.893, which is close to but slightly below the ideal 0.90 threshold. Therefore, the model fit was interpreted cautiously rather than described as unequivocally acceptable. Taken together, the fit indices suggest that the model provides a reasonable approximation of the observed data, although the NFI result indicates that model fit should not be overstated.

**Table 10:** Model fit indices.

Fit index	Estimated model	Saturated model
SRMR	0.053	0.029
d_G	0.313	0.274
Chi-square	819.077	746.982
d_ULS	0.641	0.192
NFI	0.893	0.902

The SEM analysis examined the associations among the antecedent variables, WLB, and JS. The measurement model met reliability and convergent-validity criteria, and discriminant validity was supported by the HTMT results. The structural model showed statistically significant paths from SME, ET, HDH, OLP, and PA to WLB, and from WLB to JS. The *R*^2^ values showed that the model explained 59.1% of the variance in WLB and 38.9% of the variance in JS, indicating substantial explanatory power for WLB and moderate explanatory power for JS. The *f*^2^ values further showed that OLP had the strongest antecedent-level contribution to WLB (*f*^2^ = 0.351), followed by SME (*f*^2^ = 0.270), ET (*f*^2^ = 0.238), PA (*f*^2^ = 0.211), and HDH (*f*^2^ = 0.181). The WLB → JS path showed a large effect size (*f*^2^ = 0.637), indicating that WLB made a substantial contribution to explaining JS. The positive *Q*^2^ values for WLB (*Q*^2^ = 0.374) and JS (*Q*^2^ = 0.257) indicated predictive relevance for both endogenous constructs. The bootstrapped indirect-effect results further supported WLB as a significant indirect pathway linking the antecedents with JS. The interpretation of model fit remains cautious because the NFI value was slightly below the ideal 0.90 threshold. The general summary of the SEM is provided in Table 11.

**Table 11:** Overall SEM evaluation for multi-dimensional determinants of JS model.

Category	Metric	Result	Interpretation
Measurement model	Outer loadings	0.890–0.950	All indicators above 0.70, good reliability
	VIF	2.311–4.625	No multicollinearity (VIF < 5)
	AVE	0.807–0.890	Convergent validity achieved (AVE > 0.50)
	Cronbach Alpha	0.881–0.949	High internal consistency
	CR (rho_a)	0.926–0.961	Strong construct reliability
	CR (rho_C)	0.881–0.939	Acceptable reliability
Discriminant validity	HTMT ratio	0.015–0.706	All values below 0.85, validity established
Structural paths	Path coefficient (β)	0.272–0.624	Positive relationships
	*t*-value	7.969–22.902	All values > 1.96, significant
	*p*-value	0.000	Significant at 0.001 level
Bootstrapping	Hypothesis testing	H1–H6 Supported	All paths significant after bootstrapping
Model fit	SRMR	0.053	Adequate approximate fit (< 0.08)
	NFI	0.893	Near the 0.90 threshold; interpreted cautiously
	d_ULS	0.641	Acceptable
	d_G	0.313	Acceptable
	Chi-square	819.077	Acceptable for PLS-SEM
Explanatory power	*R*^2^ for WLB	0.591	The five antecedents explain 59.1%“ of variance in WLB
	*R*^2^ for JS	0.389	WLB explains 38.9%” of variance in JS
Effect size	*f* ^2^	0.181–0.637	The f^2^ values for antecedents of WLB ranged from 0.181 to 0.351, indicating medium to large effect sizes. OLP showed the largest antecedent effect, while WLB → JS showed a large effect size.
Predictive relevance	*Q*^2^ for WLB	0.374	*Q*^2^ > 0 confirms predictive relevance. Since *Q*^2^ > 0.35, predictive relevance is large/strong.
	*Q*^2^ for JS	0.257	*Q*^2^ > 0 confirms predictive relevance. Since *Q*^2^ > .15, predictive relevance is medium.

## 5 Concluding discussion

### 5.1 Discussion of findings

Because the study used a cross-sectional, single-wave self-report design, the findings are discussed as statistically significant associations within the proposed PLS-SEM model rather than as evidence of causal effects. Terms such as “path,” “effect size,” and “indirect pathway” are used in their statistical modeling sense and should not be interpreted as demonstrating temporal or causal ordering.

**H1: Social Media Engagement and Work–Life Balance**

Hypothesis H1 predicted that SME would be positively associated with employees' perceptions of WLB. The results supported H1 and showed a positive and significant association between SME and WLB. Employees who reported higher SME also tended to report higher WLB in the present sample. This association may reflect the role of social media in helping employees stay connected with coworkers, engage in professional networking, exchange useful information, and receive emotional or informational support when managing competing work and non-work demands. According to the JD-R perspective, SME may function as a behavioral-social resource when it is used to access support and maintain professional connection. However, because of the cross-sectional design, this result should not be interpreted as evidence that SME causes improvements in WLB.

**H2: Employee Training and Work–Life Balance**

Hypothesis H2 predicted that ET would be positively associated with employees' perceptions of WLB. The findings supported H2 and showed a positive association between ET and WLB. Employees who reported more favorable training opportunities also tended to report more favorable WLB. This association may be explained by the possibility that structured training is linked with greater perceived competence, confidence, and efficiency in completing work tasks. Such resources may be associated with lower perceived work pressure and better management of work and non-work responsibilities. Nevertheless, the cross-sectional design does not allow the study to conclude that training directly improves WLB.

**H3: Healthy Dietary Habits and Work–Life Balance**

Hypothesis H3 predicted that HDH would be positively associated with WLB. The findings supported H3 and showed a positive association between HDH and WLB. Employees who reported healthier dietary habits also tended to report more favorable WLB. This association may reflect the role of health-related routines in supporting perceived energy, wellbeing, and stress regulation. However, the result should be interpreted as a statistical association rather than evidence that healthy dietary habits directly cause better WLB.

**H4: Online Learning Participation and Work–Life Balance**

Hypothesis H4 predicted that OLP would be positively associated with employees' WLB. The results supported H4 and showed that OLP had the strongest path coefficient among the antecedents of WLB. This finding can be interpreted through the association of OLP with both competence development and perceived control. Unlike many traditional training activities that are scheduled by organizations, online learning may be self-directed, modular, asynchronous, and accessible across locations. Employees who report higher OLP may therefore also perceive greater flexibility in how they develop professional skills while managing work and non-work responsibilities. Thus, the observed association may reflect not only competence-building, but also the role of perceived autonomy and flexible learning conditions. However, this result does not establish that OLP causes improved WLB; longitudinal or experimental evidence would be required to make such a claim.

**H5: Physical Activity and Work–Life Balance**

Hypothesis H5 predicted that PA would be positively associated with WLB. The findings supported H5 and showed a positive association between PA and WLB. Employees who reported higher levels of physical activity also tended to report more favorable WLB. This association may reflect the role of physical activity in stress regulation, recovery, and perceived wellbeing. However, because the data were collected at one point in time, the results cannot determine whether physical activity improves WLB, whether employees with better WLB are more able to engage in physical activity, or whether both are shaped by other unmeasured factors.

**H6: Work–Life Balance and Job Satisfaction**

Hypothesis H6 predicted that WLB would be positively associated with JS. The results supported H6, indicating that employees who reported higher WLB also reported higher JS. This finding is consistent with prior studies showing a positive relationship between WLB and JS. From the JD-R and work–life balance perspectives, employees who perceive a better balance between work and non-work responsibilities may also evaluate their job more positively. However, this association should not be interpreted as evidence that WLB directly causes higher JS. It is also possible that employees who are more satisfied with their jobs perceive their work–life balance more positively, or that both constructs are influenced by broader workplace or personal conditions.

Moreover, the bootstrapped indirect-effect results supported WLB as a significant model-based indirect pathway linking SME, ET, HDH, OLP, and PA with JS. Because the data are cross-sectional, these indirect effects should be interpreted as model-based indirect associations rather than causal mediation. Full or partial causal mediation cannot be established without longitudinal, time-lagged, or experimental data.

### 5.2 Integrative interpretation of the findings

The relative strength of the structural paths suggests that not all resources contribute to WLB in the same way. OLP showed the strongest association with WLB, which may be because online learning simultaneously addresses several modern workplace needs: continuous competence development, time flexibility, self-directed learning, and adaptation to changing job demands (Fernández-Salinero et al., 2026; Wu and Wu, 2026; Zhang et al., 2025; Xiang and Yu, 2025). SME was also positively associated with WLB, but its effect may be more conditional because digital-social engagement can function as a support resource when it facilitates communication and professional networking, while excessive connectivity may also create boundary pressure (Kumar et al., 2024; Leong et al., 2025; Pekkala et al., 2025; Fusi and Zhang, 2020). ET showed a positive association with WLB, indicating that formal training can strengthen competence and confidence, although its effect may be weaker than OLP when training is less flexible or more organization-directed (Fernández-Salinero et al., 2026; Dao et al., 2025; Arthur et al., 2026). However, because ET and OLP also showed relatively high full collinearity VIF values, their distinct contributions should be interpreted with caution, as the results may partly reflect conceptual proximity or method-related covariance between the two professional-development constructs. PA and HDH were also positively associated with WLB, suggesting that lifestyle-related personal resources support energy, recovery, and stress regulation; however, their connection to WLB may be more indirect because they operate through health and recovery mechanisms rather than through immediate work-task flexibility (Brailovskaia et al., 2024; Zagzoog et al., 2026; Dere et al., 2026; Sonnentag et al., 2023; Jackson et al., 2025). Together, these findings suggest that WLB is shaped by both workplace-centered and non-work resources, but resources that directly increase employees' perceived control over time and skill adaptation may be especially salient in contemporary work contexts (Claes et al., 2023; Zhang and Duan, 2023; Wu and Wu, 2026). These relative path strengths should be interpreted as associations estimated within the specified PLS-SEM model, not as evidence that one resource causally produces greater WLB than another.

### 5.3 Practical and policy implications

Although the cross-sectional design does not support causal intervention claims, the observed associations offer several cautious but specific implications for organizational leaders, human-resource managers, line managers, and policymakers concerned with employee wellbeing and job satisfaction. The strongest practical message is that professional-development programs should be designed as resources rather than as additional job demands. Because both ET and OLP were positively associated with WLB, organizations may benefit from supporting employee development while explicitly protecting employees from training-related overload.

First, organizations should distinguish between mandatory employee training and flexible online learning participation. Mandatory ET should be scheduled within paid working hours and integrated into workload planning so that employees are not expected to complete training on top of their normal tasks. Managers can reduce overload by temporarily adjusting deadlines, redistributing urgent tasks, or allocating protected training hours during periods of lower workload. Training modules should also be directly linked to role requirements so that employees perceive them as useful for completing work more efficiently rather than as unrelated administrative obligations.

Second, OLP should be designed around flexibility, autonomy, and pacing. Because OLP is often self-directed and asynchronous, organizations can support WLB by offering short modular courses, micro-learning units, recorded sessions, mobile-accessible materials, and realistic completion windows. Employees should be allowed to choose learning times where possible, but this flexibility should not become an expectation of after-hours study. Therefore, organizations should clearly communicate that online learning is not intended to extend the workday or replace recovery time.

Third, professional-development programs should include explicit learning-load safeguards. Before launching a training or online-learning initiative, HR departments can conduct a simple workload check to assess whether employees already face high task demands, peak operational periods, or multiple simultaneous development requirements. Organizations can also cap the number of concurrent courses, limit weekly expected learning hours, avoid clustering several mandatory modules in the same period, and provide opt-out or deferral options when employees face exceptional work or personal demands.

Fourth, line managers should be involved in translating development opportunities into feasible individual learning plans. Rather than assigning the same training schedule to all employees, managers can discuss learning goals, workload conditions, preferred learning formats, and available time with employees. Such planning may help employees benefit from competence development while maintaining control over work and non-work responsibilities. This is particularly important for OLP because flexible learning can support autonomy only when employees retain realistic control over timing and workload.

Fifth, organizations should evaluate professional-development programs not only through completion rates or test scores, but also through WLB-related indicators. Useful monitoring indicators include perceived training workload, time spent outside working hours on training, perceived usefulness of training, perceived control over learning schedules, learning-related stress, and post-training WLB. If employees report that training increases time pressure or interferes with non-work responsibilities, the program should be redesigned by reducing module length, extending deadlines, simplifying requirements, or increasing managerial support.

Finally, policy-level initiatives should encourage organizations to integrate professional development with employee wellbeing standards. Training policies should promote protected learning time, realistic workload adjustment, digital-boundary management, and voluntary participation in flexible learning where possible. In this way, professional development can be positioned not merely as a productivity tool, but as a structured resource that supports employees' competence while minimizing the risk that learning demands undermine WLB.

### 5.4 Overall conclusion

The findings indicate that professional-development, behavioral-social, and lifestyle-related personal resources are significantly associated with employees' WLB, and that WLB is strongly associated with JS. Among the antecedents, OLP showed the strongest association with WLB, followed by SME, ET, PA, and HDH. WLB also showed a strong positive association with JS. The *R*^2^ values indicated that the model explained 59.1% of the variance in WLB and 38.9% of the variance in JS, while the *f*^2^ values clarified the relative contribution of each structural path. *Q*^2^ = 0.374 for WLB indicates strong predictive relevance, whereas *Q*^2^ = 0.257 for JS indicates medium predictive relevance. These findings support the proposed resource-based framework in which professional-development, behavioral-social, and lifestyle-related personal resources are linked to job satisfaction through employees' perceived ability to balance work and non-work responsibilities. The model-fit evidence should still be interpreted cautiously: SRMR indicated adequate approximate fit, whereas NFI was slightly below the ideal 0.90 threshold.

### 5.5 Limitations and scope for future research

The findings should be interpreted with caution because this study used a cross-sectional questionnaire design with all variables collected from the same respondents at a single point in time. Therefore, the results indicate statistical relationships rather than causal effects. Although PLS-SEM was used to test the theoretically specified model, the use of terms such as path coefficient, indirect effect, and mediation refers to statistical associations within the model and should not be interpreted as proof of causality. The mediating role of WLB reflects a model-based indirect association, but causal mediation cannot be confirmed without longitudinal, time-lagged, experimental, or quasi-experimental data. In addition, because the data were collected through a single-wave self-report instrument, the results may be affected by common method variance, respondent perception bias, and unmeasured third variables. Future research should therefore use longitudinal panel designs to examine whether professional-development, lifestyle-related, and behavioral-social resources predict later changes in WLB and JS. Time-lagged designs would also help clarify the temporal ordering between antecedents, WLB, and JS.

A further limitation concerns the full collinearity VIF results used to assess potential common method bias. Although all full collinearity VIF values were below the commonly used threshold of 5, the values for OLP (VIF = 4.794) and ET (VIF = 4.733) were close to this threshold and exceeded the more conservative 3.3 criterion. This suggests that the possibility of common method variance cannot be fully excluded. In addition, because both ET and OLP represent professional-development resources, their relatively high VIF values may also reflect partial conceptual overlap or measurement redundancy between structured organizational training and flexible online learning participation. Therefore, the findings related to professional-development resources should be interpreted cautiously. Future studies should reduce this risk by using multi-source or time-lagged data, separating predictor and outcome measurement temporally, including marker variables, and developing more differentiated measurement items that clearly distinguish organization-initiated training from self-directed online learning.

Future studies should also extend the model across countries, cultures, and institutional settings to examine whether the proposed resource-mechanism framework is stable across different labor markets, digital-work cultures, and organizational policies. Multi-country studies would be particularly useful for testing measurement invariance and identifying whether the role of WLB differs across cultural and regulatory contexts. In addition, mixed-method research could strengthen the explanatory depth of the model by combining survey-based SEM with interviews or focus groups. Qualitative evidence would be especially valuable for explaining why OLP showed the strongest association with WLB and how employees experience digital learning, social-media engagement, physical activity, and dietary habits in relation to work–life boundaries.

The present study also did not test non-linear or moderated effects of SME on WLB. This is important because boundary theory and recovery theory suggest that the relationship between digital engagement and WLB may depend on use intensity, timing, perceived boundary control, after-hours availability expectations, and digital overload (Ashforth et al., 2000; Clark, 2000; Sonnentag and Fritz, 2007). Future studies should therefore examine whether SME has a curvilinear association with WLB, for example whether moderate professional-social use is beneficial whereas excessive or intrusive use becomes harmful. Future research should also test moderators such as segmentation preference, organizational digital-communication norms, recovery experiences, and perceived control over work–non-work boundaries.

Finally, future research should reduce potential common method bias by using multi-source data, supervisor-rated or organizational indicators, objective participation data from learning platforms, and more detailed behavioral measures of physical activity and dietary habits. Future SEM studies should also compare alternative direct, indirect, and partial mediation models to distinguish indirect-only pathways from full or partial mediation. Future SEM studies should further strengthen predictive evaluation by comparing alternative direct, indirect, and partial mediation models and by using longitudinal or out-of-sample predictive assessments to examine whether the proposed relationships remain stable across time and samples.

## Data Availability

The original contributions presented in the study are included in the article/supplementary material, further inquiries can be directed to the corresponding author.
